# Combining Hyperspectral Imaging with Ensemble Learning for Estimating Rapeseed Chlorophyll Content Under Different Waterlogging Durations

**DOI:** 10.3390/plants14243713

**Published:** 2025-12-05

**Authors:** Ying Jin, Yaoqi Peng, Haoyan Song, Yu Jin, Linxuan Jiang, Yishan Ji, Mingquan Ding

**Affiliations:** 1 College of Advanced Agricultural Sciences, Zhejiang A&F University, Linan, Hangzhou 311300, China; jjjjyyyy2022@163.com (Y.J.); 2024101011017@stu.zafu.edu.cn (H.S.); jy190602673@163.com (Y.J.); 202220140114@stu.zafu.edu.cn (L.J.); 2 College of Biosystems Engineering and Food Science, Zhejiang University, Hangzhou 310058, China; pengyaoqi@yeah.net

**Keywords:** waterlogging, ensemble learning, hyperspectral imaging, SPAD

## Abstract

Chlorophyll content is a key physiological indicator reflecting photosynthetic capacity, and the Soil–Plant Analysis Development (SPAD) meter is a commonly used tool for its rapid and non-destructive estimation. Hyperspectral imaging (HSI) is a non-destructive technique that captures fine spectral characteristics and thus holds great potential for high-throughput phenotyping and early stress detection. This study aimed to explore the potential of HSI combined with ensemble learning (EL) to estimate SPAD of rapeseed seedlings under different durations of waterlogging. Hyperspectral images and corresponding SPAD values were collected from six rapeseed cultivars at 0, 2, 4 and 6 days of waterlogging. The mutual information was employed to select the top 30 most relevant spectral and vegetation index features. The EL model was constructed using partial least squares, support vector machine, random forest, ridge regression and elastic net as the first-layer learners and a multiple linear regression as the second-layer learner. The results showed that the EL model showed superior stability and higher prediction accuracy compared to single models across various genotypes and waterlogging treatment datasets. As waterlogging duration increased, the overall model accuracy improved; notably, under 6 days of waterlogging, the EL model achieved an *R*^2^ of 0.79 and an RMSE of 3.27, indicating strong predictive capability. This study demonstrated that combining EL with HSI enables stable and accurate estimation of SPAD values, therefore providing an effective approach for early stress monitoring in crops.

## 1. Introduction

Rapeseed (*Brassica napus* L.), as one of the most important oilseed crops worldwide, is highly sensitive to waterlogging duration during its early growth stage [1]. Waterlogging restricts root respiration and nutrient uptake in plants, often resulting in chlorophyll degradation and a reduction in photosynthetic efficiency [2,3]. In southern China, monsoon climate and extreme weather often cause short-term waterlogging during the seedling stage of rapeseed, disrupting physiological metabolism, accelerating chlorophyll loss and ultimately reducing yield [4]. Therefore, real-time and non-destructive monitoring of chlorophyll dynamics during the waterlogging process is of great significance for early stress warning, precise stress diagnosis and the selection of waterlogging-tolerant cultivars.

Chlorophyll, a key photosynthetic pigment, converts light into chemical energy and aids in oxygen and organic compound synthesis [5]. Its content, closely linked to nitrogen nutrition and physiological activity, directly impacts crop yield and quality, making it widely used for monitoring crop growth and diagnosing stress [6]. Under abiotic stresses such as waterlogging, a reduction in chlorophyll concentration is often among the earliest physiological indicators of plant injury. Conventional chlorophyll determination methods, such as chemical extraction, although offering high analytical accuracy, are inherently destructive, time-intensive and labor-demanding, thus limiting their suitability for large-scale or high-frequency monitoring. In contrast, portable chlorophyll meters enable rapid, non-destructive measurement of soil–plant analysis development (SPAD) values which are strongly correlated with actual chlorophyll concentrations, and have been widely used in agricultural research and crop production [7,8]. However, such instruments predominantly rely on point-based measurements, which limit their capacity to capture the spatial distribution characteristics at the whole-plant or canopy scale, thereby constraining their applicability in high-throughput and large-scale monitoring scenarios.

In recent years, hyperspectral imaging (HSI) technology has advanced rapidly in the agricultural domain. This technique acquires crop reflectance across continuous narrow spectral bands and reveals vegetation physiological and biochemical status by analyzing subtle spectral variations [9,10]. Previous studies have demonstrated that hyperspectral data can be effectively utilized to achieve accurate inversion of chlorophyll content across multiple crop species. For example, Zolotukhina et al. employed acousto-optic HSI data to estimate leaf chlorophyll content with a determination coefficient of 0.89 and relative error of 15%, enabling pixel-level inversion of chlorophyll content distribution maps. [11]. Similarly, other studies have combined HSI with spectral feature processing strategies to efficiently estimate SPAD in field crops. Zhu et al. proposed a modeling approach based on hyperspectral data and spectral partitioning, which effectively estimated the spatial distribution of SPAD in saline-alkaline vegetation at a regional scale, achieving significantly higher accuracy than conventional full-sample methods [12]. Sudu et al. utilized HSI combined with multi-model comparative analysis to successfully construct a SPAD value estimation model for summer maize, achieving a maximum *R*^2^ of 0.82 [13]. These findings demonstrated that HSI offers remarkable advantages for rapid and non-destructive monitoring, providing a novel technological pathway for precision crop management under diverse environmental conditions.

Currently, most studies rely on traditional single machine learning algorithms for estimating phenotypical traits. Wang et al. reported that the random forest (RF) achieved the highest accuracy in estimating wheat biomass at the jointing, booting and flowering stages, with robustness superior to other models [14]. However, Zandler et al. found that commonly used models like stepwise regression and RF showed significant overfitting in estimating total biomass in the Eastern Pamir Mountains of Tajikistan [15]. Similarly, Ma et al. found that partial least squares (PLS) achieved the best estimation performance for SPAD across all growth stages, with the highest accuracy observed during the reproductive stage [16]. However, the findings of Yoon et al. were contrary; although PLS could be applied to predict metabolites in mustard leaves, its predictive performance was limited, with accuracy reaching only *R*^2^ = 0.53–0.58 [17]. These results indicated that the performance of a single model might vary greatly under different datasets. In recent years, ensemble learning (EL) methods have demonstrated remarkable potential in remote sensing applications such as crop physiological parameter estimation due to their superior performance. Ji et al. constructed an ensemble learning (EL) model using five base learners and a secondary learner to estimate faba bean yield and biomass. The study found that the EL model outperformed multiple individual machine learning models in both stability and accuracy, and that the fusion of multiple data sources significantly improved the model’s precision [18]. Similarly, wang et al. used four base learners and introduced a fractional-order derivative to construct a decision-level fusion (DLF) model, an implementation of EL, for detecting total nitrogen (TN) concentrations in water bodies of arid regions. Although their study found that the Gaussian Process model performed best among the individual models, it exhibited poor stability. The introduction of the DLF model significantly reduced the regression bias in the results [19]. Therefore, compared with single models, EL can effectively integrate the strengths of different algorithms, reduce the risk of overfitting, and enhance model generalization and robustness, exhibiting superior estimation performance particularly when dealing with multi-source data and complex stress conditions.

Therefore, this study employed HSI with EL to explore the temporal effects of different waterlogging durations in estimating rapeseed SPAD values. This study aimed to (1) investigate the dynamic shifts in sensitive spectral features under different waterlogging durations; (2) evaluate and compare the estimation performance of EL with basic models; (3) examine how model accuracy evolves across different waterlogging durations.

## 2. Materials and Methods

### 2.1. Experimental Design

This study selected six rapeseed varieties as experimental materials, including three conventional varieties (ZY50, ZY51, ZS11) and three hybrid varieties (HYZ50, CY45, ZYZ19). Each variety was subjected to two treatment groups: waterlogging and control. The waterlogging group was divided into three sub-groups, with waterlogging durations of 2, 4, and 6 days. All three sub-groups underwent waterlogging at the same time (with this time point serving as the baseline control, referred to as WL0). Each group of each variety was replicated eight times, totaling 144 rapeseed plants. The control group underwent no waterlogging treatment, and to eliminate spectral differences caused by plant growth, data were collected from three biological replicates at WL0 and three waterlogging time points (2, 4, and 6 days), totaling 72 rapeseed plants. All control groups were uniformly labeled as WL0. In total, 216 rapeseed plants were cultivated for subsequent experiments.

### 2.2. Data Acquisition and Processing

**Figure 1:**
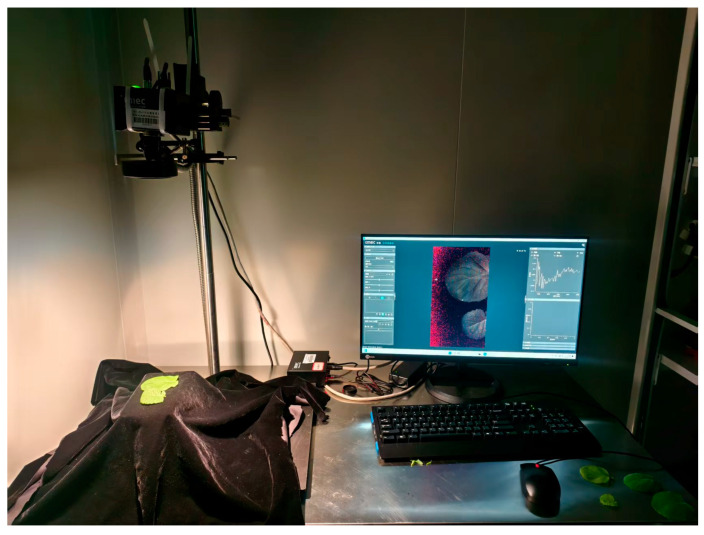
Hyperspectral image acquisition setup for rapeseed leaves.

This study utilized a portable chlorophyll meter, the SPAD-502 (Konica Minolta, Tokyo, Japan), to measure SPAD values. For each plant, four functional leaves were selected from top to bottom. Three points per leaf were sampled, and the measured values were averaged as the final values. Immediately after data collection, the leaves were placed on the imaging platform of an imec SNAPSCAN VNIR hyperspectral camera (IMEC, Leuven, Belgium) for image acquisition. The distance between the sensor and the leaves was maintained at 43 cm to ensure accurate readings (Figure 1). Prior to image acquisition, radiometric calibration was performed using a white reference panel. The exposure parameters were then optimized through an automated 10-step exposure adjustment routine to ensure optimal signal-to-noise ratio while preventing saturation. To minimize interference from ambient light and variations in temperature and humidity, imaging was conducted under dark, temperature-controlled conditions using only the instrument’s internal light source.

**Figure 2:**
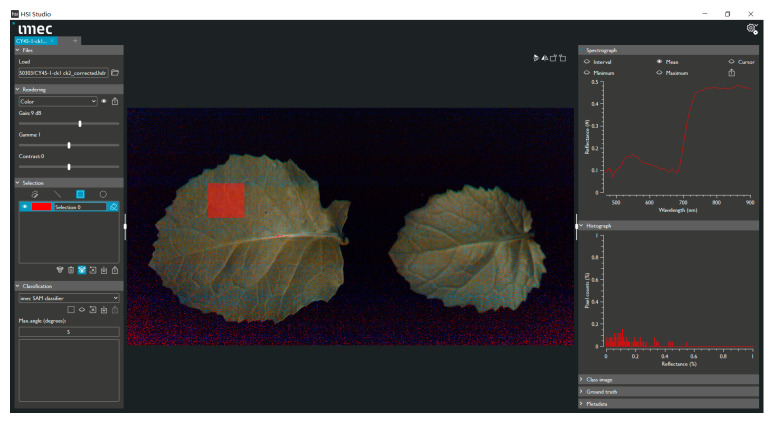
The hyperspectral image and the spectral curve of region of interest. The red region of the leaf represents the area of interest we selected. The average reflectance curve for this region is shown in the upper-right corner.

After all data were collected, HSI Studio, Version 1.2.0, IMEC, Leuven, Belgium was used to manually select the region of interest for each leaf, and all spectral data were extracted from the hyperspectral images (Figure 2).

### 2.3. Spectral and Vegetation Index Construction and Screening

The spectral measurements in this study cover a wavelength range from 470 nm to 900 nm, with a total of 150 bands. For the hyperspectral reflectance data of rapeseed seedlings obtained under each waterlogging treatment duration, a first derivative transformation was applied to reduce background noise, highlight spectral curve details and enhance spectral differences among treatments. Subsequently, reflectance values of key bands within the 445–900 nm range were extracted to calculate commonly used vegetation indices. (calculation formulas are presented in Table 1). To identify spectral features highly correlated with SPAD values, mutual information (MI) analysis was performed on both the first derivative spectra and vegetation index data for each waterlogging duration. The features were ranked in descending order of MI scores, and the top 30 were selected as input variables for subsequent regression modeling and estimation analysis. All analyses were implemented using Python version 3.10.

### 2.4. Modeling for SPAD Value Estimation

**Figure 3:**
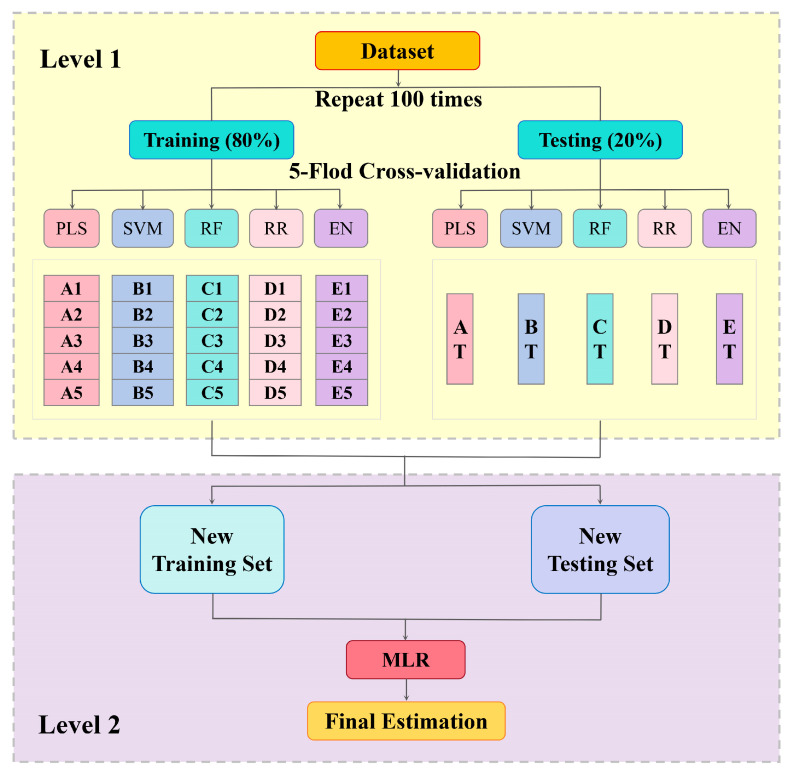
Workflow for estimating SPAD values using a stacked regression of ensemble learning model. PLS: partial least squares; SVM: support vector machine; RF: random forest; RR: ridge regression; EN: elastic net; EL: ensemble learning; MLR: Multiple Linear Regression.

This study developed an EL model based on a two-level architecture (Figure 3). In the first level, five commonly used algorithms (PLS; support vector machine, SVM; RF; ridge regression, RR; and elastic net, EN) were employed as base learners, while multiple linear regression (MLR) was used as the second level. Specifically, the original dataset was divided into training (80%) and testing (20%) subsets. The training set was further subjected to five-fold cross-validation (e.g., A1–A5, B1–B5, etc.) to optimize the parameters and evaluate the performance of the five algorithms, ensuring the robustness of model assessment. The optimized models from the training phase were then validated on the corresponding test subsets (AT, BT, CT, DT, ET). Subsequently, the estimation results from the five base algorithms were integrated to construct new training and testing sets, which were then fused by the MLR meta-learner to generate the final estimations. To enhance reliability, the entire dataset partitioning and modeling procedure was repeated 100 times with random sampling.

### 2.5. Model Training and Evaluation

In this study, model robustness was evaluated through repeated five-fold cross-validation to reduce the randomness caused by sample partitioning. The model performance was evaluated through 100 repetitions of training and testing. In each repetition, the entire dataset was randomly and independently split into a training set and a testing set. This approach ensures that the data samples used for validation are different in each run, providing a robust assessment of model generalizability. For each treatment, the top 30 features selected via MI analysis were used as input variables, and the measured SPAD values were used as output variables to construct and train each regression model. The performance metrics, including *R*^2^ and RMSE, are reported as the mean ± standard deviation across all 100 repetitions. The optimal models for different waterlogging treatments were determined based on the principle of higher *R*^2^ and lower RMSE. These metrics were calculated as follows:

(1) $$R^{2}=1-\frac{\sum_{i=1}^{n}{\left(x_{i}-\hat{x_{i}}\right)}^{2}}{\sum_{i=1}^{n}{\left(x_{i}-\bar{x}\right)}^{2}}$$

(2) $$RMSE=\sqrt{\frac{\sum_{i=1}^{n}{\left(x_{i}-\hat{x_{i}}\right)}^{2}}{n}}$$

## 3. Results

### 3.1. Statistical Analysis of SPAD Values Under Different Treatments

**Figure 4:**
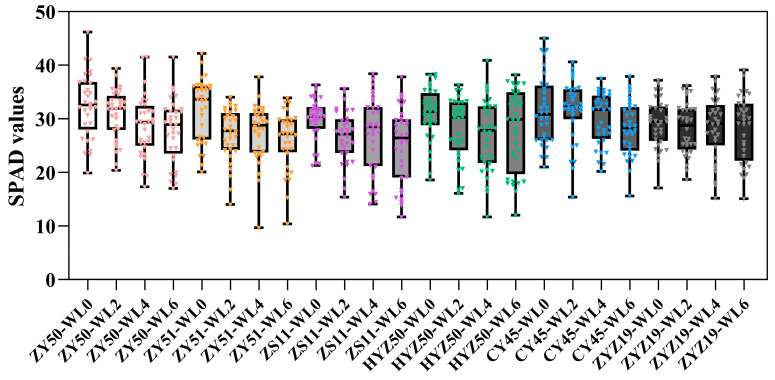
The box plots display the SPAD values for each treatment group. The whiskers represent the range of data, extending from the minimum to the maximum value within each group. The boxes represent the interquartile range (IQR), and the median is marked inside the box. WL0, WL2, WL4 and WL6 represent normal moisture conditions, 2, 4 and 6 days of waterlogging, respectively.

**Figure 5:**
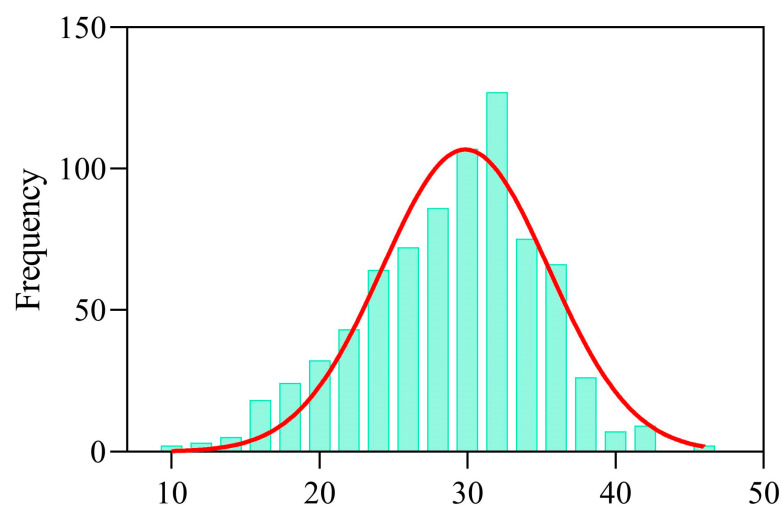
Frequency distribution histogram of the measured SPAD values.

The SPAD values of rapeseed cultivars exhibited certain varietal differences under different waterlogging durations (Figure 4). Under normal conditions (WL0), ZY50, ZY51, CY45 and HYZ50 showed higher SPAD values (30.83–32.46), which were greater than those of ZS11 (29.84) and ZYZ19 (29.20), indicating higher initial chlorophyll content in these cultivars. Overall, prolonged waterlogging led to a fluctuating decline in SPAD values. Notably, ZY51, ZS11 and ZYZ19 recorded a slightly lower SPAD value at 2 days of waterlogging (WL2) compared to 4 days of waterlogging (WL4), and HYZ50 showed a higher SPAD value at 6 days of waterlogging (WL6) compared to WL4; such fluctuations may be attributed to the random selection of sampled plants. To provide a more comprehensive view of the data characteristics, a frequency distribution analysis was performed for all measured SPAD values. The results showed that the data distribution was relatively concentrated and symmetrical, exhibiting an approximately normal distribution (Figure 5). Most values were concentrated between 20 and 40, with a peak around 30, corresponding to the highest frequency of over 100. The distribution curve gradually declined at both ends, indicating that extremely low (<15) and extremely high (>40) SPAD values occurred infrequently. These findings suggested that, under different treatments and cultivar conditions, chlorophyll content exhibited a clear central tendency while retaining a certain degree of variability, providing a solid basis for subsequent statistical analyses and model construction.

### 3.2. Screening Results of Spectral Data and Vegetation Indices

**Figure 6:**
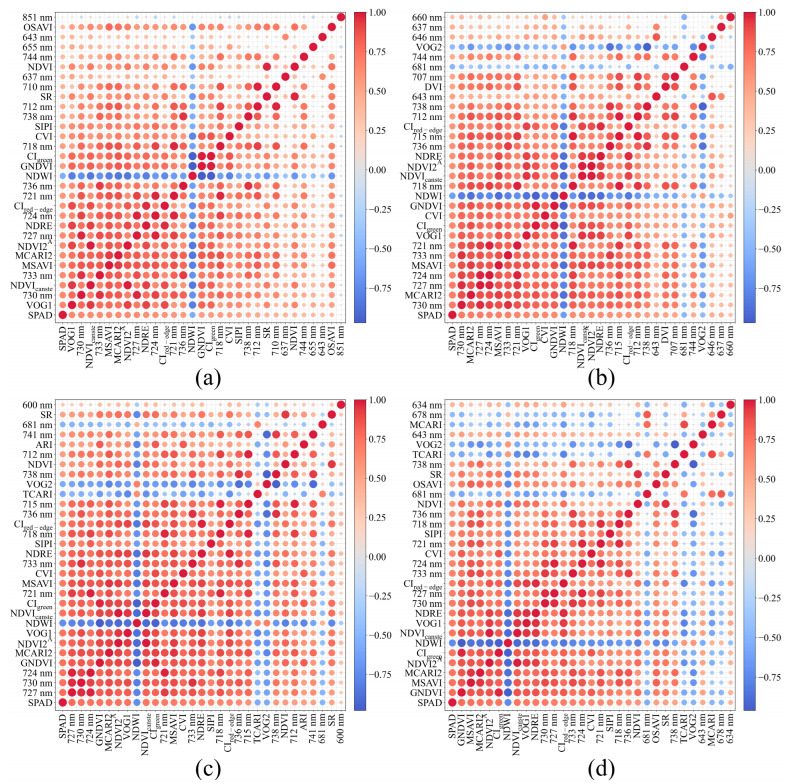
Pearson correlation matrix of SPAD values and key spectral features. (**a**): normal moisture conditions; (**b**): 2 days of waterlogging; (**c**): 4 days of waterlogging; (**d**): 6 days of waterlogging.

To investigate the relationship between measured SPAD values and hyperspectral features, the MI-based feature selection was conducted on spectral bands and vegetation indices. The 30 selected characteristic variables were then subjected to a Pearson correlation analysis with the measured SPAD values. The results were shown in Figure 6.

In this study, the correlation analysis between rapeseed SPAD values and hyperspectral features under various waterlogging durations revealed that chlorophyll content exhibits significant response patterns in the red-edge and near-infrared spectral regions. Under WL0 (Figure 6a), a strong positive correlation was observed between SPAD and several features, including VOG1 (r = 0.65), 730 nm (r = 0.64), NDVI_canste_ (r = 0.64), 733 nm (r = 0.63), MCARI2 (r = 0.63) NDVI2^A^ (r = 0.63) and 727 nm (r = 0.62). Concurrently, NDWI (r = −0.548) exhibited a significant negative correlation. After WL2 (Figure 6b), the features with strong positive correlations were primarily concentrated in the NIR region, including 730 nm (r = 0.71), MCARI2 (r = 0.71), 727 nm (r = 0.71), 724 nm (r = 0.68), MSAVI (r = 0.68), 733 nm (r = 0.68), 721 nm (r = 0.66) and VOG1 (r = 0.65). The main negatively correlated features were NDWI (r = −0.64), 681 nm (r = −0.47) and VOG2 (r = −0.45). At WL4 (Figure 6c), the positive correlations were further enhanced. The highest correlation coefficients were found for the 727 nm (r = 0.76), 730 nm (r = 0.75) and 724 nm (r = 0.74) bands, with indices such as GNDVI (r = 0.74), MCARI2 (r = 0.74), NDVI2^A^ (r = 0.74) and VOG1 (r = 0.74) also showing significant positive correlations. The primary negative correlators were NDWI (r = −0.74), TCARI (r = −0.56), VOG2 (r = −0.56) and 681 nm (r = −0.50). Under WL6 (Figure 6d), GNDVI (r = 0.77) exhibited the highest correlation, followed by MSAVI (r = 0.76), MCARI2 (r = 0.75), NDVI2^A^ (r = 0.74), CI_green_ (r = 0.74), NDVI_canste_ (r = 0.73), VOG1 (r = 0.72) and several other NIR bands and related indices. The negative correlations remained concentrated on NDWI (r = −0.74), 681 nm (r = −0.53), TCARI (r = −0.50), VOG2 (r = −0.49), MCARI (r = −0.46) and 678 nm (r = −0.44). With prolonged waterlogging, the sensitive features gradually extended from the red-edge to the near-infrared region, and the correlations became increasingly stronger, thereby providing a solid foundation for subsequent regression model development.

### 3.3. Estimation Performance of Six Different Models

After feature selection using MI, the 30 features selected for each waterlogging duration were separately input into the six different models.

**Figure 7:**
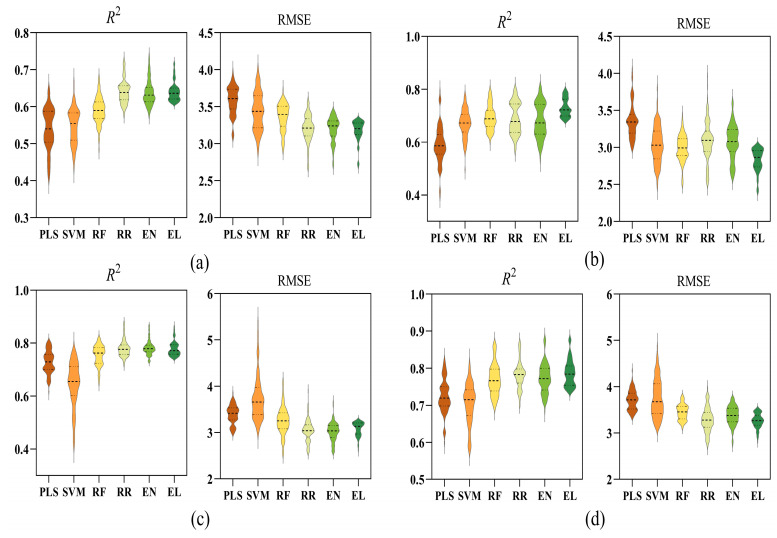
Comparison of *R*^2^ and RMSE for different SPAD estimation models. (**a**): normal moisture conditions; (**b**): 2 days of waterlogging; (**c**): 4 days of waterlogging; (**d**): 6 days of waterlogging. PLS: partial least squares; SVM: support vector machine; RF: random forest; RR: ridge regression; EN: elastic net; EL: ensemble learning. Dashed lines depict the distribution: the darker line marks the median, and lighter lines bound the interquartile range (25th–75th percentiles).

Figure 7 presents the differences in six models (PLS, SVM, RF, RR, EN and EL) in estimating SPAD values under different waterlogging durations (0, 2, 4 and 6 days). Under WL0, the models exhibited a clear performance hierarchy. The EL model demonstrated a leading advantage, with the highest *R*^2^ (0.64 ± 0.03) distributions among all models and the lowest RMSE (3.18 ± 0.14) distributions. Notably, although RR and EL achieved similar overall performance metrics, the EL model exhibited a narrower performance distribution (as indicated by the violin plot in Figure 7a), suggesting greater consistency and stability in its estimations. The EN model followed closely, while PLS, SVM and RF performed comparatively worse. Under WL2, the predictive performance of the models continued to show clear differences (Figure 7b). Overall, the EL model performed optimally on both the *R*^2^ (0.72 ± 0.03) and RMSE (2.83 ± 0.15) metrics. Its *R*^2^ value distribution was the highest and most concentrated, while its RMSE value was the lowest with the least fluctuation, indicating that it possesses both high accuracy and excellent stability. RR and EN continued to maintain good performance, with relatively high *R*^2^ and low RMSE values. The performance of RF was slightly lower than that of RR and EN, while SVM and PLS performed relatively poorly. Notably, the mean value of *R*^2^ for PLS was 0.59 ± 0.70 with a large range of fluctuation, and its RMSE (3.35 ± 0.23) distribution was also high. Under WL4, RR, EN and EL continued to maintain high performance levels in terms of *R*^2^ (0.78 ± 0.03) (Figure 7c). Among them, the EN model had a lower RMSE (3.02 ± 0.15), indicating superior estimation stability. The RF model’s performance was moderate, while the *R*^2^ values for the PLS and SVM models were significantly lower with greater fluctuation. Notably, SVM exhibited the widest distribution, indicating poor stability. In terms of RMSE, the mean values for the EN and RR models were lower than those of the EL model; however, the EL model’s data distribution was more concentrated, demonstrating the best stability. Under the WL6 treatment, the models still showed notable performance differences (Figure 7d). The EL model showed the best overall performance, with the highest mean value of *R*^2^ (0.79 ± 0.04) and the most concentrated distribution, indicating both high accuracy and strong stability. It also achieved the lowest RMSE (3.27 ± 0.16), confirming minimal estimation error. RR ranked second, while EN and RF performed at intermediate levels. In contrast, PLS and SVM had lower goodness of fit (*R*^2^ = 0.70–0.73), wider indicator distributions, and higher RMSE values (≥3.8), suggesting poorer stability and accuracy under this stress stage.

### 3.4. Comparison of Six Models Under Different Waterlogging Durations

**Figure 8:**
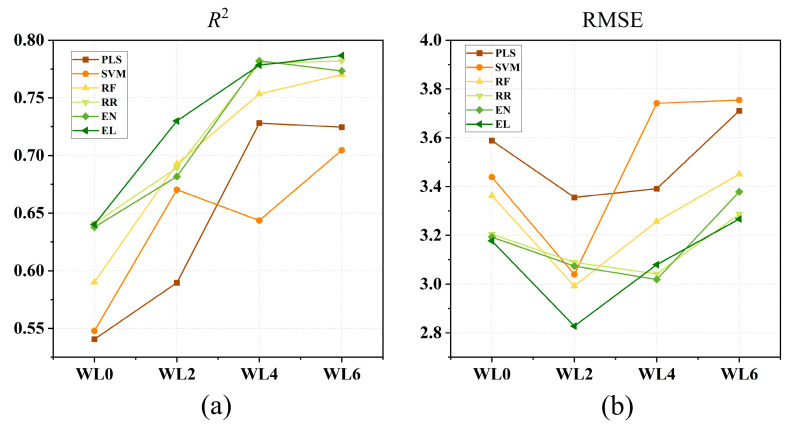
(**a**,**b**) Mean *R*^2^ and RMSE of each model under different waterlogging durations. WL0, WL2, WL4 and WL6 correspond to normal moisture conditions, 2, 4 and 6 days of waterlogging, respectively. PLS: partial least squares; SVM: support vector machine; RF: random forest; RR: ridge regression; EN: elastic net; EL: ensemble learning.

To provide a unified comparison of model performance changes across different waterlogging durations, a line graph of their performance was plotted (Figure 8).

Figure 8 illustrates the estimation performance variations in six models (PLS, SVM, RF, RR, EN and EL) under different waterlogging durations. Overall, the EL model demonstrated the most robust performance, with its *R*^2^ increasing consistently from 0.64 ± 0.03 at WL0 to 0.79 ± 0.04 WL6, while maintaining the lowest RMSE values (3.18 ± 0.14 at WL0, 2.83 ± 0.15 at WL2, 3.08 ± 0.15 at WL4, and 3.27 ± 0.16 at WL6), indicating high accuracy and low estimation error. The EN and RR models followed closely, with *R*^2^ comparable to EL. Specifically, RR (3.04 ± 0.23) and EN (3.02 ± 0.20) achieved a lower RMSE than EL at WL4. Although the RF model exhibited somewhat lower *R*^2^ (0.55 ± 0.05 at WL0 to 0.72 ± 0.05 at WL6) and slightly higher RMSE (2.99–3.45), it remained competitive. In contrast, the PLS (0.54 ± 0.06) and SVM (0.55 ± 0.05) models showed the lowest initial *R*^2^, and their RMSE remained above 3.60 during WL4 and WL6, indicating relatively poor estimation reliability under prolonged stress.

In summary, the EL model delivered the best overall performance, combining high accuracy, low error and stable behavior. The EN and RR models also performed strongly, ranking as competitive alternatives. The RF model achieved moderate results, while the PLS and SVM models lagged in both predictive accuracy and robustness.

### 3.5. Effect of Waterlogging Durations on Model Performance

**Figure 9:**
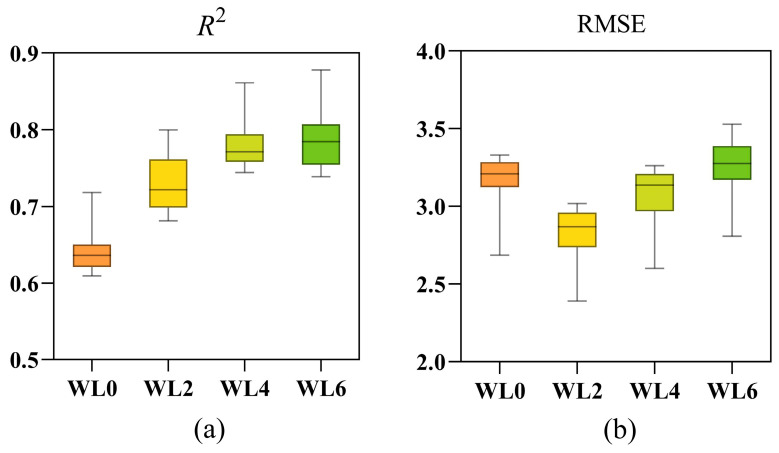
(**a**,**b**) Box plots of the *R*^2^ and RMSE for the EL model at four waterlogging durations. WL0, WL2, WL4 and WL6 correspond to normal moisture conditions, 2, 4 and 6 days of waterlogging, respectively.

In order to further explore the influence of waterlogging durations on model performance, the EL model was adopted for subsequent analysis. The results are illustrated in Figure 9.

As shown in Figure 9a, the *R*^2^ gradually increased with the duration of waterlogging. The model exhibited the lowest performance under WL0, with a median *R*^2^ of 0.61. As waterlogging progressed, the accuracy of models improved significantly. The median *R*^2^ surpassed 0.70 at WL2 and approached 0.80 at WL4. The highest performance was observed at WL6, where the median *R*^2^ exceeded 0.80, and the narrower interquartile range indicated greater model stability and higher estimation accuracy under prolonged stress. In contrast, the RMSE, as depicted in Figure 9b, showed a “U-shaped” trend. The model’s estimation error was highest at WL0, with a median RMSE of 3.20. The error then decreased significantly at WL2 (median RMSE of 2.87), indicating optimal estimation performance. Subsequently, the RMSE values increased at WL4 and WL6, ultimately returning to a level similar to that of the control group, suggesting a decline in the model’s predictive stability under long-term waterlogging.

Overall, the EL model demonstrated superior performance in estimating SPAD values under different waterlogging durations compared to normal growing conditions. It is noteworthy that while the model achieved its highest goodness-of-fit (*R*^2^) at WL6, its lowest estimation error (RMSE) was observed at WL2.

### 3.6. Model Training Results of Rapeseed Leaves Under Full-Cycle Waterlogging Stress (WLS)

**Figure 10:**
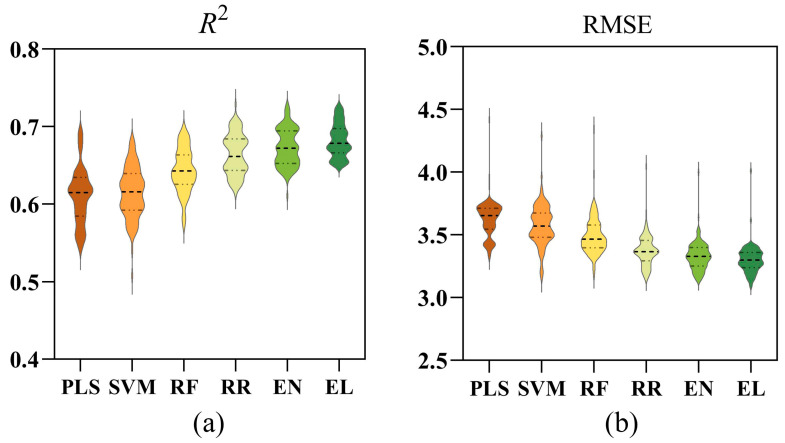
(**a**,**b**) Comparison of *R*^2^ and RMSE for different SPAD estimation models. PLS: partial least squares; SVM: support vector machine; RF: random forest; RR: ridge regression; EN: elastic net; EL: ensemble learning.

Figure 10 shows the results of modeling with data integrated from all WLS stages. In terms of *R*^2^ (Figure 10a), the EL model performs the best with an average value of 0.68, followed closely by EN, RR, and RF with average *R*^2^ values of 0.67, 0.66, and 0.64, respectively, demonstrating strong fitting capabilities. In contrast, PLS and SVM show slightly lower *R*^2^ values, averaging 0.61. Regarding RMSE (Figure 10b), all models exhibit relatively low errors, ranging between 3.0 and 4.0, with PLS and SVM having higher RMSE values of 3.63 and 3.58, respectively. RF performs at a moderate level, while RR and EN have lower RMSE values around 3.5. The EL model achieves the smallest error, with an average RMSE of 3.30. Overall, the EL model performs better and is more stable.

### 3.7. Prediction Performance of Six Rapeseed Cultivars Under Different Models

**Figure 11:**
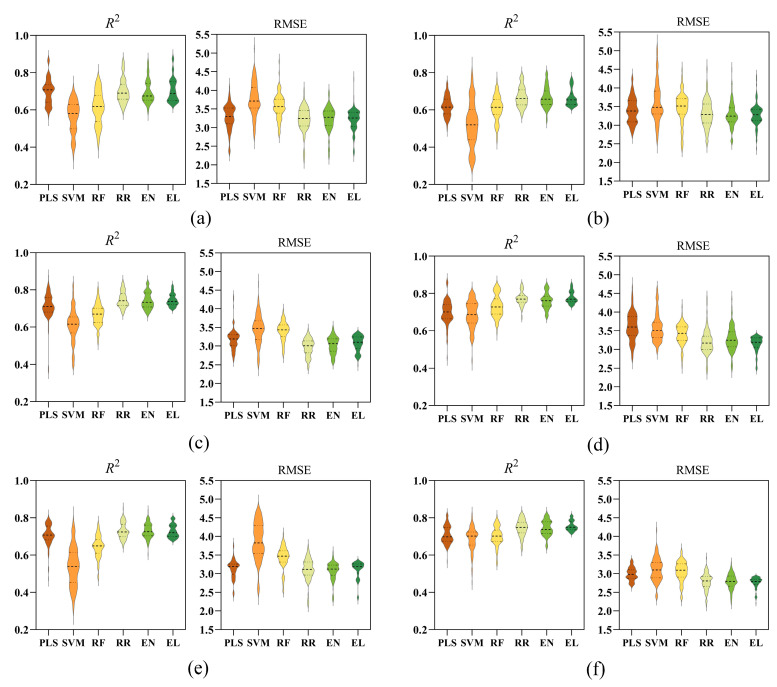
*R*^2^ and RMSE of different SPAD estimation models for the six varieties. (**a**): ZY50; (**b**): zy51; (**c**): ZS11; (**d**): HYZ50; (**e**): HYZ50; (**f**): HYZ50. PLS: partial least squares; SVM: support vector machine; RF: random forest; RR: ridge regression; EN: elastic net; EL: ensemble learning. PLS: partial least squares; SVM: support vector machine; RF: random forest; RR: ridge regression; EN: elastic net; EL: ensemble learning.

Figure 11 presents the prediction performance of the six evaluated models across the six rapeseed cultivars. Notable cultivar-dependent variations in model efficacy were observed. The EL model demonstrated superior predictive performance for cultivars ZY50, ZYZ19, and HYZ50, achieving *R*^2^ values of 0.71, 0.76, and 0.78, respectively, coupled with the lowest RMSE values, indicating high accuracy and stability. The RR model also exhibited strong performance, particularly for cultivar ZS11, where it attained an *R*^2^ of 0.75 and an RMSE of 2.97. Both EL and RR models maintained robust predictive capabilities for cultivars CY45 and ZY51. In contrast, the SVM model consistently underperformed across all cultivars, as evidenced by lower *R*^2^ and higher RMSE values, indicating limited predictive ability.

These findings suggest that while the RR model shows strong adaptability to specific cultivars, the EL model provides the most robust, accurate, and reliable predictions across a diverse set of genotypes. Therefore, the EL model is identified as the most suitable and dependable choice for estimating SPAD values under waterlogging stress in rapeseed.

## 4. Discussion

Chlorophyll content is a key indicator of plant health under WLS. Numerous studies have utilized HSI to estimate chlorophyll content in crops [41]. However, research on estimating chlorophyll content in rapeseed under varying waterlogging durations is currently lacking, and establishing a quantitative relationship between waterlogging durations and physiological damage is crucial for early warning [42,43]. This study evaluated the feasibility of combining HSI with EL for the non-destructive estimation of early WLS in rapeseed. By selecting key spectral and vegetation indices and constructing a stacked EL model, the results indicated that the EL model achieved high accuracy and stability across most stress durations, with performance improving as the duration of stress increased. Overall, the integration of EL and HSI provides a stable, precise and effective approach for early crop stress monitoring and high-throughput phenotyping.

Spectral information and its derived vegetation indices are closely related to plant traits [44]. The results of this study showed that the correlation patterns between rapeseed leaf SPAD values and hyperspectral features changed significantly with the duration of WLS. Under mild stress conditions (WL0, WL2), features positively correlated with SPAD are primarily concentrated in the narrow bands near the red-edge (727–733 nm) and red-edge-based vegetation indices (MSAVI, MCARI2), which is consistent with the strong chlorophyll absorption and high reflectance characteristics in this region [45]. As waterlogging duration increased (WL4, WL6), the wavelength range of positively correlated features gradually expanded from the narrow red-edge bands to gradually expanding to composite indices combining green light and red-edge, such as GNDVI and CIgreen. This change suggested that the complex physiological reorganization induced by stress environments may contribute to this: for example, chlorophyll degradation and changes in pigment composition regulating visible light absorption, collapse of mesophyll structure weakening light scattering, and water loss adjusting near-infrared reflectance. The combined effects of these factors likely lead to a higher dependency on multi-band combinations for estimating SPAD values under stress conditions. Liu et al. (year) reported that the spectral bands most sensitive to changes in leaf water content under WLS are located in the visible and short-wave infrared regions [46]. It is worth noting that water stress significantly affects the reflectance characteristics of leaves in the visible light spectrum, particularly in the green light region. Therefore, spectral changes induced by water deficit, combined with the previously mentioned chlorophyll degradation effect in the green light region, collectively drive the expansion of SPAD-sensitive features towards composite indices of green light and red-edge. This mechanism is especially pronounced during the later stages of stress (WL4 and WL6): indices such as GNDVI, MCARI2, NDVI2A, and CIgreen, which utilize both green light and red-edge information, show correlation coefficients with SPAD exceeding 0.70. This confirms that under complex stress, the synergistic information from water-sensitive bands (green light) and chlorophyll-sensitive bands (red-edge) is crucial for chlorophyll estimation. Conversely, NDWI, defined using Green-NIR, consistently shows a significant negative correlation with SPAD across all stress durations, reaching its strongest at WL4 and WL6 (r = −0.74). As Marusig et al. [47] showed, NDWI tracks leaf water content, and during waterlogging, the increase in NDWI is accompanied by a decrease in SPAD, indicating an inverse correlation between the two. In summary, the correlation between different bands and vegetation indices with SPAD evolves dynamically with the duration of stress: in the early stages, it is dominated by single red-edge bands and red-edge indices, while in the later stages, it expands to composite indices combining green light and red-edge. At the same time, negative correlations with features like NDWI remain significant throughout. This finding provides a reference for constructing chlorophyll monitoring models based on hyperspectral data that are adapted to different stages of stress.

The predictive capabilities of different model structures for SPAD values vary significantly depending on the data. Previous research has shown that EL algorithms possess high accuracy and stability in plant nutrient diagnosis [48,49]. In this study, the performance of six regression models in predicting rapeseed leaf SPAD values under different waterlogging durations varied significantly and exhibited a clear temporal evolution pattern. Overall, EL model demonstrated a higher *R*^2^ and lower RMSE across all waterlogging stages, with a concentrated performance distribution, indicating its superior predictive accuracy as well as better stability and generalization ability. In terms of genotype adaptability, the EL model proved most effective, maintaining high stability and accuracy across multiple cultivars (e.g., ZY50, ZYZ19, HYZ50). This contrasts with the RR model, showing strong cultivar-specific performance (notably on ZS11), attributed to its strong regularization capability, but less versatile, and the SVM model, which performed poorly across all varieties [50]. This suggests that while the EL model offers the best generalization, future work could focus on developing highly tailored, cultivar-specific models to achieve even greater precision.

The superior performance of the EL model across all waterlogging durations stems from its dual capability to achieve the lowest RMSE in early stress stages (WL0-WL2), ideal for sensitive detection, while also yielding the highest *R*^2^ (approaching 0.80) during severe stress (WL4-WL6), reflecting its robust fitting capacity. This highlights a critical finding: the divergence between the prediction error (RMSE), observed at the mild WL2 stage, and the maximum model fit (*R*^2^), which occurred during severe stress. We attribute this phenomenon to a shift in the underlying spectral drivers; mild stress is dominated by relatively simple changes in leaf water status, leading to low prediction error, whereas prolonged stress introduces more complex physiological alterations like chlorophyll degradation. While these latter changes have a stronger overall correlation with SPAD (higher *R*^2^), their complexity increases the prediction error (RMSE). This inherent complexity also explains why a model trained on aggregated data from all stages performed sub-optimally, as mixing physiologically distinct signals introduces noise and compromises accuracy.

Although this study demonstrates the high accuracy and stability of the HSI and EL models in predicting chlorophyll content under controlled waterlogging conditions, the transferability of these models to multiple cultivars and real-world field environments remains a challenge. The number of variety types and environmental factors such as light variation, canopy structure, and soil reflectance, can affect plant spectral characteristics, thus influencing model performance. For instance, changes in outdoor lighting conditions may lead to variations in leaf spectral reflectance, which could differ significantly from the controlled laboratory settings. Additionally, canopy structure and soil reflectance may introduce extra noise, affecting the amount of light reflected from the leaves and the surrounding environment. These factors can reduce the generalization ability of the models. On the other hand, although SPAD values were used as the primary reference in this study, the lack of validation with actual chlorophyll content data means that SPAD is a relative measurement. Future work will focus on field validation by collecting spectral data from various field environments, including different plant cultivars, growth stages, and environmental conditions. This will help assess the robustness and adaptability of the models under real-world conditions. Future research will focus on validating SPAD values against actual chlorophyll content using chemical analysis or spectroscopic techniques to improve model accuracy. Moreover, domain adaptation strategies, such as transfer learning, will be explored to adapt models trained on controlled data to real-world scenarios.

## 5. Conclusions

This study systematically evaluated the potential of combining HSI with EL for estimating rapeseed chlorophyll content under different waterlogging durations. The conclusions are summarized as follows:

(1) Prolonged WLS expanded sensitive features from the red-edge to green-red-edge areas and intensified its inverse correlation with the water index NDWI.
(2) The EL model showed greater stability than single models when applied to various data from genotypes and WLS treatments. It also demonstrated higher prediction accuracy in most models.
(3) EL model achieved its highest goodness-of-fit (*R*^2^) at the WL6, but its lowest prediction error (RMSE) in WL2.

This study demonstrated that the integration of HSI and EL provides a robust technological pathway for high-throughput crop phenotyping and precision agricultural management. This combined approach offers a highly accurate and stable method for the early detection of WLS in rapeseed.

## Figures and Tables

**Table 1:** Vegetation indices and calculation formula.

Vegetation Index	Calculation Formula	Reference
Anthocyanin Reflectance Index	$\mathrm{ARI}=(1/R_{500})-(1/R_{700})$	[20]
Chlorophyll Index	${\mathrm{CI}}_{\mathrm{green}}=(R_{800}/R_{550})-1$	[21]
	${\mathrm{CI}}_{\mathrm{red}-\mathrm{edge}}=(R_{800}/R_{720})-1$	[21]
Chlorophyll Vegetation Index	$\mathrm{CVI}=(R_{800}/R_{550})\times (R_{670}/R_{550})$	[22]
Difference Vegetation Index	$DVI=R_{810}\;-\;R_{680}$	[23]
Green Normalized Difference Vegetation Index	$GNDVI=(R_{800}\;-R_{550})/(R_{800}+R_{550})$	[24]
Modified Soil Adjusted Vegetation Index	$\mathrm{MSAVI}=0.5\times (2\times R_{800}+1-{\left({(2\times R_{800}+1)}^{2}-8\times {(R}_{800}-R_{700})\right)}^{0.5})$	[25]
Modified Simple Ratio	$\mathrm{MSR}=(R_{800}/R_{670}-1)/{(R_{800}/R_{670}+1)}^{0.5}$	[26]
Modified Chlorophyll Absorption Ratio Index	$\mathrm{MCARI}=\left(\left(R_{700}-R_{670}\right)-0.2\times \left(R_{700}-R_{550}\right)\right)\times \left(R_{700}/R_{670}\right)$	[27]
	$\mathrm{MCARI}2=\left(\left(R_{750}-R_{705}\right)-0.2\times \left(R_{750}-R_{550}\right)\right)\times \left(R_{750}/R_{705}\right)$	[28]
Moisture Stress Index	$\mathrm{MSI}=R_{860}/R_{800}$	[29]
Normalized Difference Water Index	$\mathrm{NDWI}=(R_{560}-R_{860})/(R_{560}+R_{860})$	[30]
Normalized Difference Red Edge Index	$\mathrm{NDRE}=(R_{790}-R_{720})/(R_{790}+R_{720})$	[31]
Normalized Difference Vegetation Index	$NDVI=(R_{800}-R_{680})/(R_{800}+R_{680})$	[32]
	${\mathrm{NDVI}2}^{A}=(R_{750}-R_{705})/(R_{750}+R_{705})$	[33]
	${\mathrm{NDVI}}_{canste}=(R_{760}-R_{708})/(R_{760}+R_{708})$	[33]
Optimized Soil Adjusted Vegetation Index	$\mathrm{OSAVI}=1.16\times \left(R_{800}-R_{670}\right)/\left(R_{800}+R_{670}+0.16\right)$	[34]
Photochemical Reflectance Index	$\mathrm{PRI}=(R_{531}-R_{570})/(R_{531}+R_{570})$	[35]
Ratio Vegetation Index	$RVI=R_{800}/R_{670}$	[36]
Renormalized Difference Vegetation Index	$RDVI=(R_{800}-R_{670})/{{(R}_{800}+R_{670})}^{0.5}$	[32]
Simple Ratio	$SR=R_{800}/R_{680}$	[37]
Structure Insensitive Pigment Index	$\mathrm{SIPI}=(R_{800}-R_{445})/(R_{800}-R_{680})$	[38]
Transformed Chlorophyll Absorption Ratio Index	$\mathrm{TCARI}=3\times \left(\left(R_{700}-R_{670}\right)-0.2\times \left(R_{700}-R_{550}\right)\right.\times \left.\left(R_{700}/R_{670}\right)\right)$	[39]
Triangular Vegetation Index	$\mathrm{TVI}=0.5\times \left(120\times \left(R_{750}-R_{550}\right)-\left.200\times \left(R_{670}-R_{550}\right)\right)\right.$	[39]
Vogelmann Index	$\mathrm{VOG}1=R_{740}/R_{720}$	[40]
	$\mathrm{VOG}2=\left(R_{734}-R_{747}\right)/\left(R_{715}+R_{726}\right)$	[40]

Note: The hyperspectral data was collected in the range from 470 nm to 900 nm, with a spectral resolution of approximately 2.8–3.0 nm. All vegetation indices were calculated by extracting the single-band reflectance closest to the theoretical wavelength values from the dataset. The wavelengths in the data have decimal values but are close to the theoretical values with minimal error. For example, for the PRI index, we selected 530.6040 nm and 571.0067 nm as the wavelengths.

## Data Availability

The original contributions presented in this study are included in the article. Further inquiries can be directed to the corresponding author.
